# Adsorption of Methylene Blue on PVDF Membrane and PVDF/TiO_2_ Hybrid Membrane: Batch and Cross-Flow Filtration Studies

**DOI:** 10.3390/polym18020233

**Published:** 2026-01-16

**Authors:** Fengmei Shi, Boming Fan, Shuqi Ma, Hao Lv, Chao Lin, Jin Ma, Wei Jiang, Yuxin Ma

**Affiliations:** 1 Heilongjiang Academy of Black Soil Conservation and Utilization, Heilongjiang Academy of Agricultural Sciences, Harbin 150086, China; ocean-water@126.com; 2 College of Civil Engineering, Heilongjiang University, Harbin 150080, China; 3 Research and Development Centre, Shandong Aisen Water Industry Co., Ltd., Taian 271021, China

**Keywords:** PVDF/TiO_2_ hybrid membrane, methylene blue, adsorption mechanism, Dubinin–Radushrevich equation, Thomas model

## Abstract

The adsorption of methylene blue (MB) on poly(vinylidene fluoride) (PVDF) and PVDF/titanium dioxide(TiO_2_) membranes with 1.5 wt% dosage was examined through batch adsorption and dynamic cross-flow filtration experiments. The effects of pH, temperature, and initial MB concentration on adsorption performance were evaluated via batch experiments. The Thomas model was applied to analyze the membrane filtration process, while kinetic, isothermal, and thermodynamic models were integrated to elucidate the adsorption mechanisms. Results demonstrated that low temperature and high initial MB concentration significantly improved MB adsorption on both membranes. Under neutral pH conditions (pH = 7), the maximum adsorption capacities of PVDF and PVDF/TiO_2_ membranes reached 1.518 ± 0.025 mg/g and 0.189 ± 0.008 mg/g, respectively. The adsorption processes on both membranes conformed to the pseudo-second-order kinetic model, with optimal fitting to the Langmuir isotherm model. Thermodynamic analysis revealed physical adsorption mechanisms, as evidenced by adsorption free energy (*E*) calculated via the Dubinin–Radushrevich model Notably, PVDF membrane exhibited a more pronounced mass transfer zone height (*h*_Z_ = 2.3 ± 0.1 cm) and achieved higher adsorption capacity (2.1 ± 0.09 mg/g) than PVDF/TiO_2_ membranes (0.25 ± 0.01 mg/g). The TiO_2_ incorporation reduced hybrid membrane adsorption capacity and significantly mitigated membrane fouling caused by adsorption, with PVDF/TiO_2_ membranes showing a 32 ± 2.5% lower flux decline rate than PVDF membranes with less MB into the pores. This study provides fundamental data supporting the combined application of “adsorption–subsequent oxidation” using PVDF-based membranes in dye wastewater treatment.

## 1. Introduction

Wastewater containing dyes is difficult to degrade in aerobic treatment systems and toxic to microorganisms, due to the presence of functional groups such as phenyl, azo, and amino groups [1,2]. Among these compounds, methylene blue (MB), a typical aromatic cationic dye with a molecular weight of 319.85 Da, poses a significant threat to aquatic ecosystems and human health when present in excess [3]. A range of physical, chemical, and biological methods have been employed to address dye pollution [4]. Adsorption, with its simplicity, cost efficiency, high efficacy, and broad applicability [5], is viewed as a potentially effective solution for dye removal from wastewater. Recent research has largely focused on low-cost adsorbents such as biosorbents [6,7,8,9,10], biochar [11], carbon nanotubes [12], clay [12,13], Fe-Mn, Si, and Ti oxides [5,14,15], activated carbon [16,17,18,19,20], polymer membrane [21], and anaerobic granular sludge [22]. However, these options may present limitations including slower adsorption rates, reduced adsorption capacity, and suboptimal reusability [23,24]. Therefore, there is a pressing need to develop novel recyclable adsorbents or innovative technologies to navigate these challenges. Some studies have explored MB desorption by modulating solution pH values, but this approach has proven incomplete, necessitating further re-treatment or decomposition of the desorbed MB. A more promising strategy involves the oxidation and decomposition of MB molecules through ultraviolet (UV) treatment or advanced oxidation treatment paired with catalysts [25,26]. Photocatalytic oxidation via an aqueous nano-TiO_2_ suspension shows particular promise for MB photodegradation, although the need for nano-TiO_2_ particle recycling increases the costs [27,28,29,30].

A poly (vinylidene fluoride) (PVDF) membrane has several advantages including an open pore structure, high mechanical strength, and chemical stability which makes it a promising polymer carrier for incorporating TiO_2_ nanoparticles [31,32], but it suffers from fouling because of the hydrophobic nature of PVDF molecules. The integration of TiO_2_ nanoparticles into the polymeric membrane matrix could improve the anti-fouling performance and create superior photo-degradation properties [33]. The UV catalytic properties of PVDF/TiO_2_ hybrid membranes were discovered as having superior photocatalytic degradation ability in MB solution compared to a neat PVDF membrane [30]. However, despite their enhanced photo-catalytic properties, the catalytic degradation of MB took longer, limiting the technique’s application in membrane filtration. These catalytic mixed-matrix membranes were ultrafiltration membranes with a dense skin layer, resulting in lower adsorption capacities. In a previous study, we prepared an isotropic (no skin layer) PVDF/TiO_2_ membrane with higher porosity and strength using the thermally induced phase separation (TIPS) method, which is essential for the catalytic process and water treatment [34]. While investigating their photocatalytic performance during filtration, we observed the strong adsorption of MB by the membranes (accounting for >85% of total removal). Although this phenomenon interfered with photocatalysis experiments, it suggested a potential application strategy involving “adsorption enrichment followed by oxidative degradation”.

Existing studies have primarily focused on the photocatalytic performance of PVDF/TiO_2_ membranes, with limited systematic analysis of their adsorption mechanisms. These studies exhibit the following shortcomings: (1) the mechanism by which TiO_2_ affects the adsorption sites of PVDF remains unclear; (2) the mass transfer characteristics under dynamic filtration conditions have not been integrated with batch experiments; and (3) there is a lack of quantitative data regarding the synergistic relationship between adsorption and antifouling properties.

This study employs batch and cross-flow filtration experiments to compare the MB adsorption behavior of neat PVDF membranes and PVDF/TiO_2_ membranes, aiming to address the following key issues: (1) clarify the dominant mechanism of MB adsorption by PVDF (hydrophobic interactions vs. electrostatic interactions); (2) quantify the effects of TiO_2_ on membrane adsorption capacity, mass transfer efficiency, and antifouling properties; and (3) identify the critical factors enhancing the adsorption performance under dynamic filtration conditions, providing a scientific basis for the application of PVDF-based membranes in dye wastewater treatment.

## 2. Materials and Methods

### 2.1. Materials

The flat sheet neat PVDF and PVDF/TiO_2_ membranes were fabricated using the TIPS method, as detailed in our prior study [34]. The neat PVDF (PT-0) membrane was prepared with 30 wt% PVDF (FR904, $\bar{M_{n}}$ = 380,000, Shanghai 3F New Materials Co., Ltd., Shanghai, China) and 70 wt% Dimethyl Phthalate (DMP, Shanghai Bio Life S&T. Co., Ltd., Shanghai, China). Conversely, the PVDF/TiO_2_ (PT-1.5) membrane was prepared with 28.5 wt% PVDF with 1.5 wt% TiO_2_ (Anatase, Meidilin Nanometer Material Co., Ltd., Zhongshan, China, average diameter 20 nm) and 70 wt% DMP. The basic parameters of the membranes are shown in Table S1 (Supplementary File S1), where the pure water flux of the PT-0 membrane was 63.4 ± 1.3 L·m^−2^·h^−1^ with a contact angle of 108 ± 2.4°; the pure water flux of the PT-1.5 membrane was 86.3 ± 3.2 L·m^−2^·h^−1^ with a contact angle of 119.5 ± 2.4°. Bovine Serum Albumin (BSA, *M*w = 67,000 Da) was employed for the membrane fouling tests, while methylene blue (MB, or tetramethylthionine chloride) was sourced from Shanghai Bio Life Science & Technology Co., Ltd. (Shanghai, China).

### 2.2. Adsorption Studies

#### 2.2.1. Batch Adsorption

The membrane samples with approximately the same weight for the same batch of trials were cut into approximately 1 mm × 1 mm pieces and then used as adsorbents. The initial MB concentration used in this study was determined according to the relevant literature [30]. At a high MB concentration, in order to shorten the test cycle, more adsorbents were used in the isothermal adsorption and pH effect tests, according to the equilibrium time results. Meanwhile, it was difficult to strictly keep the same thickness of membranes. So, the weight of membrane/hybrid membrane samples used in the experiment varied from 1.6 to 2.2 g/L for different MB concentrations. These pieces were placed into 250 mL glass flasks containing 100 mL of MB solution at varying concentrations, and the adsorption of MB by the glass containers was ignored during the experiment. The pH values of the MB solutions were adjusted using 0.1 mol/L HCl and/or NaOH solutions. Subsequently, the flasks were secured in a thermostatic orbital shaker at a speed of 30–40 r/min and shaken for a specified duration. Finally, the MB concentration in each flask was determined using a UV-spectrophotometer at a wavelength of 665 nm. All the experiments were performed 3 times. The experiments were conducted under the following conditions:

(1) Determination of the equilibrium time: the dosage of the adsorbent (membrane/hybrid membrane samples) was set at 1.6 g·L^−1^; the initial MB concentration (MB_0_) was 3.68 mg·L^−1^; the pH was maintained at 7.0 ± 0.1; the shaking time varied from 0 to 180 min at different intervals (with shorter intervals at the onset of adsorption); the temperature was kept at 25 ± 0.1 °C.
(2) Isotherm adsorption studies were conducted with an adsorbent dosage of 2.0 ± 0.1 g·L^−1^, an initial concentration of MB (MB_0_) ranging from 0 to 80 mg·L^−1^, a pH of 7.0 ± 0.1, and a shaking time of 120 min at a temperature of 25 ± 0.1 °C.
(3) Effect of Temperature: the study was conducted with an adsorbent dosage of 1.7 ± 0.1 g·L^−1^, an initial concentration of MB (MB_0_) at 3.20 mg·L^−1^, a pH level of 7.0 ± 0.1, a shaking time of 120 min, and a temperature range of 10–40 °C.

#### 2.2.2. Dynamic Adsorption

The PT-0 and PT-1.5 membranes were selected to investigate the dynamic adsorption of MB in a cross-flow filtration system (Figure S2, Supplementary File S3) using a circulating MB solution [35]. To prevent the accumulation of the retained solutes, the permeate solution was redirected to the initial solution via the outlet three-port valve. A portion of the solution in the bypass flow was collected every 10 min for analysis. As the common operating pressure of microfiltration membrane is 0.1 MPa, in order to simultaneously remove the MB in water during the filtration process, the operating pressure of the cross-flow filtration was maintained at 0.1 MPa in this study. The relationships between the filtrate concentration, retentate concentration, permeate flux, and adsorption capacity (*q*) with respect to the permeate volume (*V*) were determined for both membrane samples. The experiments were performed 3 times.

## 3. Results and Discussion

### 3.1. Kinetics Study

**Figure 1:**
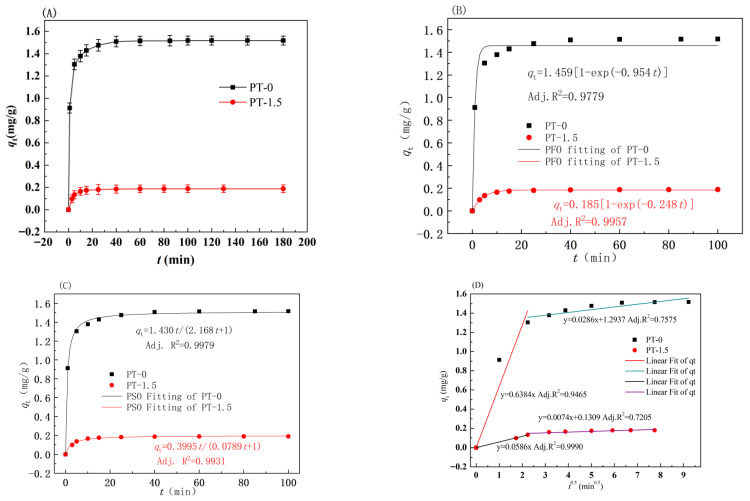
Effect of contact time on MB adsorption (**A**), the PFO kinetic model (**B**), the PSO kinetic model (**C**), and intra-particle diffusion model (**D**) for batch adsorption (*T* = 25 °C, adsorbent dosage: 1.6 g·L^−1^, *C*_0_ = 3.68 mg·L^−1^).

Figure 1A shows that the adsorption of MB by both membranes exhibited the fastest rate within the initial 10 min (the PT-0 reached an adsorption capacity of 1.37 ± 0.05 mg/g, accounting for 90.2% of the equilibrium amount; the PT-1.5 reached 0.17 ± 0.01 mg/g, accounting for 89.9% of the equilibrium amount), with an equilibrium time of 100 min for both (no significant change in adsorption capacity was observed when extended to 180 min; hence, 100 min was selected as the equilibrium time). The equilibrium adsorption capacity of the PT-0 membrane (1.518 ± 0.025 mg/g) was significantly higher than that of the PT-1.5 membrane (0.189 ± 0.008 mg/g), consistent with the photographs of the membranes after adsorbing MB (PT-0 appeared deep blue, and PT-1.5 appeared light blue; see Supplementary File S3, Figure S2). Among the common dyes, the anionic dye of methyl orange (MO, *M*w = 327.33) and the cationic dye of MB (*M*w = 319.85) have similar molecular weights. The adsorption capacity of MO by the PVDF composite membranes at 3 mg·L^−1^ of the initial concentration of MO was reported to be 3.5, 4.8, 2.2, and 4.7 mg·g^−1^ with 0, 0.13%, 13.33%, and 26.32% of TiO_2_ addition, respectively [36]. The 1-bromohexadecane-grafted glycidyl methacrylate PVDF membrane exhibited higher flux and could remove 99.25% of MB, 99.69% of MO, and 99.39% of neutral-red at the initial MB concentration of 100 mg·L^−1^ [37]. The possible reason is that the decrease in the active sites of the membranes resulted from the aggregation of inorganic nanoparticles or the interaction between inorganic nanoparticles and PVDF molecules, which prevented the adsorption of MO on the membranes [36,37]. The adsorption area of the PVDF/TiO_2_ membrane increased by 8.26%, according to the results calculated in Supplementary File S4. Assuming the adsorptive capacity of titanium dioxide for MB was negligible, the calculated equilibrium adsorption capacity of PVDF in the hybrid membrane was 0.20 mg·g^−1^. This value represents 13% of the actual equilibrium adsorption capacity of the pristine PVDF membrane, based on the mass ratio of PVDF to the hybrid membrane. Furthermore, it was observed that the adsorbed MB exhibited high stability within the membrane material and displayed resistance to desorption into water. This behavior underscores the strong binding affinity of MB to the membrane. There are adsorption activation sites within PVDF, which interact with MB molecules to a certain degree. The incorporation of nano-TiO_2_ into the membrane reduces the equilibrium adsorption capacity, suggesting that the nano-TiO_2_ particles may occupy the PVDF’s adsorption activation sites and inhibit the adsorption of MB on the membrane materials. Furthermore, the data suggest interactions between PVDF and TiO_2_. As detailed in Table S2 (Supplementary File S5), first-principles Molecular Dynamics (MD) calculations revealed interaction/binding energies between PVDF and TiO_2_, originating from electrostatic and van der Waals forces. These forces could also enhance the antifouling performance of novel hybrid membranes. The occupation of adsorption activation sites on the membranes by nanomaterials would reduce the irreversible adsorption of foulants.

Several kinetic models are typically employed to elucidate the governing mechanism of the adsorption process [38,39,40]. The pseudo-first-order (PFO) and pseudo-second-order (PSO) non-linear equations are often used to study the kinetic process and presented as Equation (1) and Equation (2), respectively [41,42,43]:

(1) $$q_{t}=q_{e}\cdot [1-\exp(-k_{1}\cdot t)],$$

(2) $$q_{t}=\frac{q_{e}^{2}\cdot k_{2}\cdot t}{\left[k_{2}\cdot q_{e}\cdot t+1\right]},$$

where *q*_e_ and *q*_t_ are the amount of MB adsorbed (mg·g^−1^) at equilibrium and at *t* (min), respectively, *k*_1_ (min^−1^) is the adsorption rate constant of PFO adsorption, and *k*_2_(g·mg^−1^·min^−1^) is the correlation coefficient for the PSO kinetic equation.

The correlation coefficients for the PT-0 membrane, as determined in Figure 1B via the PFO kinetic model, are relatively low, with a significant deviation between the calculated *q*_e_(cal) and experimental *q*_e_(exp) values, as shown in Table 1. The adjusted *R*^2^ value of the PSO model for the PT-0 membrane (0.9979) was significantly higher than that of the PFO model (0.9779), and the calculated equilibrium adsorption capacity (q_e,cal_ = 1.516 ± 0.009 mg/g) deviated from the experimental value (*q*_e_(exp) = 1.518 ± 0.025 mg/g) by only 0.1%. This indicates that the adsorption of MB onto the membrane may be governed by chemical processes, due to the negatively charged surface of the PVDF membrane [44]. For the PT-1.5 membrane, both the PFO and PSO models showed high adjusted *R*^2^ values (0.9957 and 0.9931, respectively), but the deviation between the *q*_e,cal_ value of the PSO model (0.197 ± 0.003 mg/g) and the experimental value was smaller (4.2%). This indicates that the adsorption of MB on both membranes is controlled by the inter-molecular forces (such as the surface site binding caused by hydrophobic interactions [42]. Similar phenomena have been observed in the adsorption of MB on coir pith carbon [45], perlite [46], and wheat shells [47], as well as in dye adsorption on activated carbon [48,49]. The increase in the contact angle of PT-1.5 membrane was much higher than that of PT-0, which means more hydrophobicity and unfavorability for the adsorption of water-soluble substances, leading to lower a MB adsorption capacity than for the PT-0. At the same time, TiO_2_ in the surface of the membrane would interact with MB and PVDF, resulting in a different ratio of physical adsorption to chemical adsorption than that of PT-0 [50]. When the concentration of MB in the solution is constant, the adsorption process is controlled by the above factors; so, the adsorption process can be modeled by the PSO and PFO kinetic equations, and both have high correlation coefficients. The *q*_e_(cal) values obtained by the PSO model and PFO model are similar to the *q*_e_(exp) values of the PT-1.5 membrane.

The model for intra-particle diffusion is presented in Equation (3), serving to delineate the diffusion mechanism prevalent during the adsorption process [50].

(3) $$q_{t}=k_{p}t^{1/2}+M,$$

where *q*_t_ (mg·g^−1^) is the amount of MB adsorbed at time t (min), *k*_p_ is the intra-particle diffusion rate constant (mg·g^−1^·h^−0.5^), and M is the intercept.

The intraparticle diffusion model fitting results (Figure 1D) indicate that the adsorption process can be divided into different stages: the transient surface adsorption of MB on the membrane surface (0–10 min) and the diffusion of MB into the membrane pores until adsorption equilibrium within the pores (10–100 min). The fitted curve is not a linear fit, indicating that intraparticle diffusion is not the sole rate-controlling step [51]. The suboptimal performance in the initial simulation of the PSO kinetic model and intra-particle diffusion model may be attributed to two factors: the excessively rapid adsorption rate of MB by the PT-0 membrane and the structural and performance differences between the PT-0 and PT-1.5 membranes that result in distinct interactions with the MB molecules.

### 3.2. Isotherm Adsorption

The Langmuir and Freundlich models are often used as isotherm adsorption models. The adsorption mechanisms of MB on the surfaces of PT-0 and PT-1.5 membranes are examined using the Langmuir and Freundlich isotherm models, represented as Equation (4) and Equation (5), respectively [39,40].

(4) $$q_{e}=\frac{Q_{0}K_{L}C_{e}}{1+K_{L}C_{e}},$$

(5) $$q_{e}=K_{f}C_{e}^{1/n},$$

where, *Q*_0_ (mg·g^−1^) and *K*_L_ (L·mg^−1^) are the Langmuir constants. *K*_f_ ((mg·g^−1^)(L·mg^−1^)^1/n^) and 1/n are the Freundlich constants. Figure S4 (Supplementary File S6) illustrates the isothermal adsorption of MB on both the PT-0 and PT-1.5 membranes at varying temperatures, while Table 2 displays the parameters derived from the isothermal models.

Figure S4 illustrates that as the MB concentration increases, the quantity of adsorbed MB molecules on the membrane surfaces also rises. This can be attributed to the increased driving force of the concentration gradient when the concentration of MB is elevated. Upon comparing the *R*^2^ values, it can be concluded that the Langmuir isotherm model provides a more accurate description of the adsorption behavior of MB on both PT-0 and PT-1.5 membranes. The Langmuir model fitting results (Figure S4A, Supplementary File S6) demonstrated that the maximum adsorption capacity (*Q*_0_ = 14.481 ± 3.994 mg/g) of the PT-0 membrane at 288 K (15 °C) was higher than that at 298 K (25 °C) (*Q*_0_ = 8.997 ± 0.284 mg/g). The active adsorption sites of the PT-0 membranes are more uniform than those of the PT-1.5 membranes, as indicated by the adj. *R*^2^ values. The PT-1.5 membrane exhibits a lower adsorption capacity than the neat PVDF membrane, suggesting that the incorporation of nano-TiO_2_ particles into the PVDF membrane diminishes the adsorption sites.

The FTIR analysis of both membranes is shown in Figure S5 (Supplementary File S7). The characteristic peak at 1186 cm^−1^ indicates the -CF_2_ stretching of PVDF [36,52]. The absorption peaks of the C=S stretching vibration (1730 cm^−1^) and the C=N stretching vibration in infrared absorption (1210 cm^−1^) all indicate the presence of the MB structure [53,54]. In the FTIR spectra of PT-0 MB and PT-1.5 MB, characteristic infrared peaks of C=N and C=S stretching vibration can be found, indicating that MB molecules are absorbed by both membranes. PT-0 and PT-0 MB have similar characteristics of peaks to PT-1.5 and PT-1.5 MB, respectively. No new peaks could be found in the FTIR diagram of the membranes before and after adsorption, indicating there is no chemical adsorption. It may be that the size exclusion effect of TiO_2_ will block the MB molecules into membrane pores and make the PVDF/TiO_2_ membrane have good anti-fouling performance [30].

The Dubinin–Radushrevich isotherm model provides insights into the adsorption mechanism and can be articulated as [55]:

(6) $$\ln X=\ln X_{m}-K\varepsilon^{2},$$

where *X* and *X*_m_ are the adsorption amount and adsorption capacity of MB adsorbed on adsorbent (g·g^−1^), respectively, *K* is the constant associated with the adsorption energy, *C* is the MB concentration at equilibrium (g·L^−1^), and *R* and *T* are the gas constant (kJ·K^−1^·mol^−1^) and the absolute temperature (K), respectively.

The average adsorption free energy (*E*) is employed to ascertain the type of adsorption and is represented as

(7) $$E={\left(-2K\right)}^{-0.5}.$$

**Figure 2:**
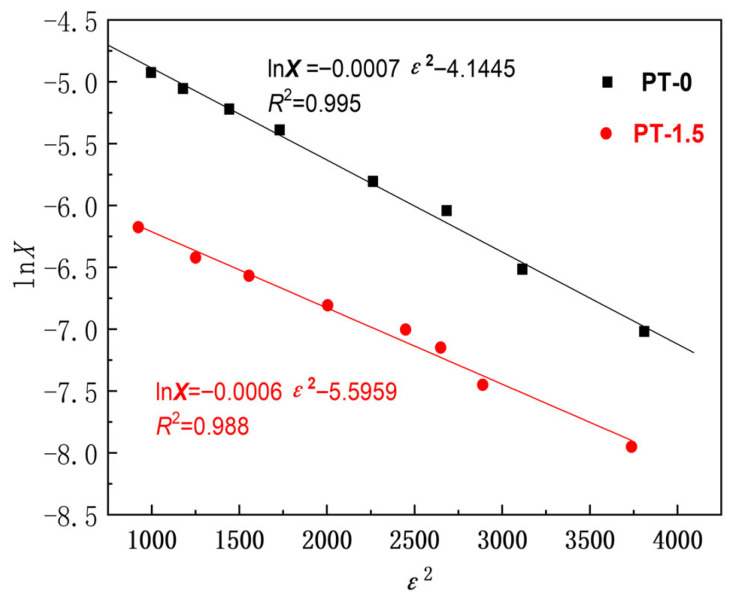
Analysis of Dubinin–Radushrevich kinetic model.

The graphical representation of lnX in relation to *ε*^2^ is depicted in Figure 2, with parameters such as *K*, *X*_m_, and *E*, as detailed in Table 3. The adsorption free energy E (Table 3) calculated by the DR model in Figure 2 (PT-0: 26.73 ± 0.52 kJ/mol, PT-1.5: 28.87 ± 0.61 kJ/mol) exceeds the conventional physical adsorption threshold of E < 8 kJ/mol [56,57]. The organic membrane adsorbing dye molecules may be due to the H-bonding, π-π stacking interactions, electrostatic interactions, ionic interactions, hydrophobic interactions, van der Waals’s interactions, and pore-filling [58,59]. The adsorption mechanism is much more complex than the interpretation of the Dubinin–Radushrevich isotherm model.

The introduction of TiO_2_ reduces the adsorption capacity of the PT-1.5 membranes due to three factors: (1) the addition of TiO_2_ changes the selectivity of the PT-1.5 membrane on MB molecules [50]; (2) the presence of TiO_2_ decreases the membrane pore size from 75 nm (PT-0) to 65 nm (PT-1.5), increasing the diffusion resistance of MB (Supplementary File S2, Figure S1); (3) the interactions between the PVDF matrix and TiO_2_. could affect the diffusion, adsorption, and blocking behavior of MB molecules [58].

### 3.3. Effect of pH Values

**Figure 3:**
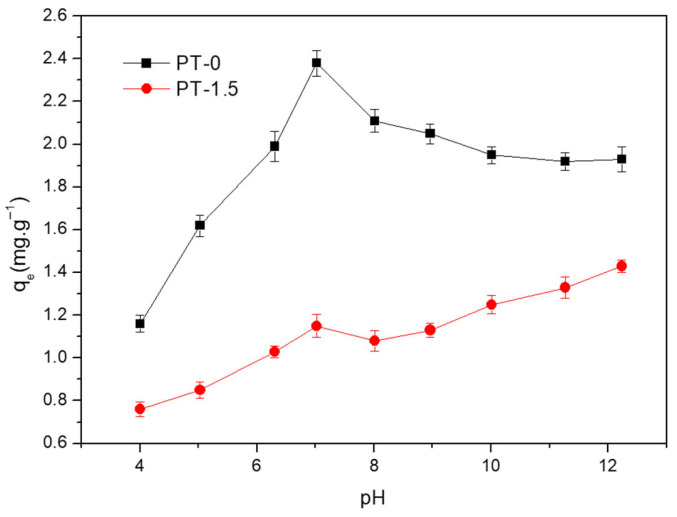
Effect of pH value on the adsorption capacities of MB.

The pH value of the solution can affect the properties of the adsorbent and adsorbate, bringing about changes in the interactions between the adsorbent and adsorbate. Figure 3 shows that one peak of adsorption capacities for both membranes can be found at pH = 7, and the adsorption capacities of PT-1.5 are much less than that of PT-0. The adsorption capacity of the adsorbent varied with the pH value. It is reported that the pH can enhance or hinder the protonation process of dye and obtain different adsorption capacities [59]. When pH < 7, the high concentration of H^+^ in solution competed with MB^+^ for adsorption sites on the membrane surface, and the TiO_2_ underwent protonating, leading to a decreasing trend and difference in adsorption capacities; (2) when pH > 7, fewer MB molecules exist as the cation form of C_16_H_18_N_3_SCl^+^, which could be favorable to be absorb to the membrane. The deduction of these functions would lead to the MB^+^ undergoing hydrolysis to form neutral NOH groups (pKa = 7.2), reducing its hydrophobicity, while Ti-OH on the TiO_2_ surface converted to Ti-O^−^, weakening the interaction with PVDF (zeta potential decreased from −8.2 mV to −15.6 mV shown in Supplementary File S8). However, the reduction in MB’s hydrophobicity dominated the change in the adsorption capacity of the PT-0 membrane, resulting in an overall decreasing trend [60]. The sustained increase in the adsorption capacity of the PT-1.5 membrane at pH values significantly above 8 is particularly noteworthy. The reasons should be studied further. The pH appears to modulate the electrostatic interactions between the PVDF or PVDF/titanium dioxide membranes and MB [61]. The reasons should be studied further. The results hint that it is better to undergo the adsorption or separation process under appropriate pH conditions.

### 3.4. Effect of Temperature

**Figure 4:**
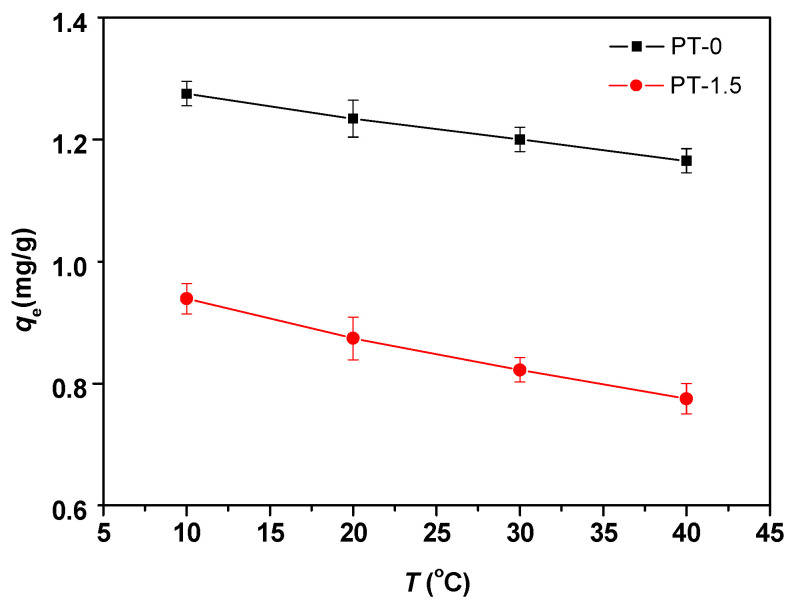
Effect of temperature on the adsorption capacities of MB for the PT-0 membrane and PT-1.5 membrane.

As depicted in Figure 4, a decrease in temperature elevates the equilibrium adsorption capacity. At higher temperatures, MB molecules exhibit increased mobility, enabling them to readily detach from the membrane pore surface.

The feasibility and favorability of adsorption are reflected in the thermodynamic parameters, including the free energy change (∆*G*^⊖^), enthalpy change (∆*H*^⊖^), and entropy change (∆*S*^⊖^). These parameters can be estimated using Equation (8) (Supplementary File S9) [62,63,64].

ln*K*_d_ = ∆S^⊖^/*R* − ∆*H*^⊖^/(*RT*) (8)

The ∆*G*^⊖^ values are computed utilizing Equation (S4). The thermodynamic parameters’ values are displayed in Table 4. Negative ∆*G*^⊖^ values suggest that the adsorption process is both feasible and spontaneous under standard conditions.

The ∆*H*^⊖^ values for the PT-0 or PT-1.5 membranes at different temperatures are 188.0 kJ·mol^−1^ and 140.8 kJ·mol^−1^, respectively, indicating an endothermic adsorption process. These ∆*H*^⊖^ and ∆*S*^⊖^ values for both types of membranes surpass those reported for other adsorption processes. This could be attributed to the high porosity and large capillary pore structure of the membrane, which promotes capillary adsorption. The increased porosity and additional adsorption sites contribute to the superior adsorption capacity of the PT-0 membrane, thereby elevating ∆*H*^⊖^ and ∆*S*^⊖^. However, when nano-TiO_2_ is dispersed within the membrane, the resulting decrease in membrane porosity and uniform pore size, coupled with the nano-TiO_2_ adsorption effect, reduces the adsorption capacity, leading to lower ∆*H*^⊖^ and ∆S^⊖^ values. The presence of potential surface hydroxyl groups and broken bonds elevates the atomic energy of the nanoparticle surface compared to that of internal atoms. This allows surface atoms of the particles to combine with other atoms, enhancing adsorption. The highly developed pore structure of the PT-0 membrane, characterized by its strong adsorption capacity and expansive surface area, facilitates comprehensive contact between the MB molecule and the capillary. This leads to the adsorption of the MB molecule by the fluorine atoms on the capillary wall due to their electronegativity. Once the MB molecule is adsorbed into the capillary, the mutual attraction between MB molecules attracts additional molecules until the capillary is saturated.

The ∆*G*^⊖^ values for both PT-0 and PT-1.5 membranes are negative, indicating that the adsorption process is thermodynamically favorable and spontaneous [63]. The temperature increase is beneficial to the spontaneity of the adsorption process, but according to the results of the adsorption experiment, the adsorption capacity is reduced at the same time. This could be attributed to the increased temperature of the solution enhancing the tendency of MB molecules to escape from the solid phase to the bulk liquid phase [63]. MB molecules are adsorbed on the surface of capillary pores of the membrane. The mobility of MB molecules is low at low temperature; so, the adsorption capacity is high. Generally, the ∆*G*^⊖^ for physisorption and chemisorption is between −20 ~ 0 kJ/mol and −80 ~ −400 kJ/mol, respectively [65,66]. The ∆*G*^⊖^ of the MB adsorption on either PT-0 or PT-1.5 membranes falls within the range of −30 to −15 kJ·mol^−1^, suggesting that the adsorption process is strong physical adsorption, which is consistent with the results of the DR model [66].

### 3.5. Dynamic MB Adsorption in a Cross-Flow Filtration

**Figure 5:**
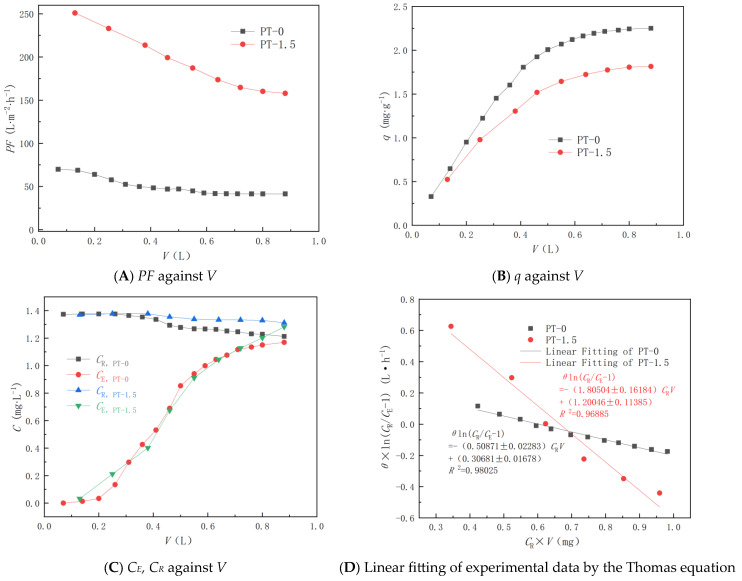
Filtration experiment of MB solution under the trans-membrane pressure of 0.1 MPa.

Figure 5 depicts the changes in filtrate concentration (*C*_E_), retentate concentration (*C*_R_), permeate flux (*PF*), and adsorption capacity (*q*), as they relate to the permeate volume (*V*) for two membrane samples during the filtration experiment of an MB solution under a trans-membrane pressure of 0.1 MPa.

Figure 5A demonstrates that the permeation flux of the PT-1.5 membrane surpasses that of the PT-0 membrane under equivalent operational trans-membrane pressure. This superior performance is attributed to the enhanced hydrophilicity and anti-compaction capabilities [30]. As filtration progresses, the adsorption of additional MB molecules obstructs membrane pore channels, consequently diminishing the permeation flux. For the PT-0 membrane, the MB fouling layer forms, and the pure water flux becomes stable much faster than PT-1.5 does before the water flux is measured. Hence, the water flux of PT-1.5 decreases faster than that of PT-0. With the increase in the residence time, more MB molecules will diffuse to the membrane surface to form a contamination layer and block the membrane pores until the thickness of the contamination layer remains unchanged. Figure 5B illustrates that MB adsorption capacity escalates in tandem with the permeate volume, with the PT-0 membrane exhibiting superior adsorption capacity compared to the PT-1.5 membrane. This is due to the PT-0 membrane offering a larger number of active adsorption sites. Consequently, as filtration continues, more MB molecules are retained at the PVDF adsorption sites, thereby augmenting the MB adsorption capacity. Figure 5C depicts a gradual reduction in *C*_R_ over time, attributable to the dilution of the recycled permeate solution. The *C*_R_ for the PT-0 membrane declines more rapidly, reflecting its enhanced adsorption capacity. Furthermore, the *C*_E_ for both membranes escalates with an increase in the permeate volume. The relationship between *C*_E_ and *V* resembles an S-curve, akin to a breakthrough curve observed in column adsorption experiments, and the data can be analyzed using the linear form of the Thomas equation [67].

(9) θ ln(CRCE−1)=kTqTm−kTCRV,

where *C*_R_ and *C*_E_ are the retentate concentration and the permeate concentration (mg·L^−1^), respectively, *k*_T_ is the rate constant (L·mg^−1^·h^−1^), *θ* is the filtration rate (L·h^−1^), *q*_T_ is the total adsorption capacity (mg·g^−1^), *V* is the permeate volume (L), and m is the mass of the membrane (g).The height of the mass transfer zone (*h*_Z_) can be calculated by the following equation [68].

(10) $$h_{Z}=\frac{H\left(V_{E}-V_{B}\right)}{\left[V_{E}-\left(1-f\right)\left(V_{E}-V_{B}\right)\right],}$$

where *H* is the membrane thickness (cm), and *f* is the fraction of membrane still able to remove MB. The *f* can be defined as

(11) $$f=\int_{0}^{1}\left(1-\frac{C}{C_{R}}\right)d\left[\frac{\left(V-V_{B}\right)}{\left(V_{E}-V_{B}\right)}\right]=\int_{V_{B}}^{V_{E}}\frac{\left(C_{R}-C\right)dV}{C_{R}(V_{E}-V_{B}),}$$

where *V*_B_ is the permeate volume at breakthrough point(L), and the other variables have been defined previously.

Figure 5D illustrates the linear fit of the experimental data, specifically utilizing the data from between the breakthrough point at *C*_E_ = 5% *C*_R_ and the exhaustion point at *C*_E_ = 95% *C*_R_, to the Thomas equation. The PT-0 membrane exhibits a sharper breakthrough S-curve due to its shorter *h*_Z_ in filtration and decreasing filtration velocity. Conversely, the curve for the PT-1.5 membrane is less pronounced, as the membrane’s thickness is less than the *h*_Z_ in filtration. The PT-1.5 membrane displays a higher *k*_T_ value, suggesting that the mass transport resistance within the membrane is reduced. This can be attributed to the enhanced hydrophilicity of the membrane pore surface facilitated by nano-TiO_2_.

The adsorption capacity of MB in the PT-0 membrane is notably higher than that of the PT-1.5 membrane, a finding that aligns with the outcomes of the previously discussed batch adsorption. The cross-flow filtration process demonstrates a superior MB adsorption capacity compared to the batch adsorption process. This enhancement can be attributed to the operative pressure on the permeation flow, which aids in the mass transfer process of MB molecules, thereby enabling their ready diffusion to the inner pore surface of the membrane. Hence, although PT-0 membrane has higher porosity and lower CA than the PT-1.5 membrane, its flux is much lower than that of PT-1.5 because less MB is adsorbed on the PT-1.5 membrane surface, which blocks the pores. Furthermore, the experiment suggests that the incorporation of TiO_2_ can mitigate membrane fouling due to adsorption. Compared with the adsorption capacity of composite membranes in the other studies (Table S3 in Supplementary File S10), the adsorption capacity of the PVDF/TiO_2_ membrane is much lower, which has good antifouling properties and indicates the prospective applications of these PVDF/TiO_2_ composite membranes in the purification of dye wastewater.

### 3.6. Proposed Adsorption Mechanism

**Figure 6:**
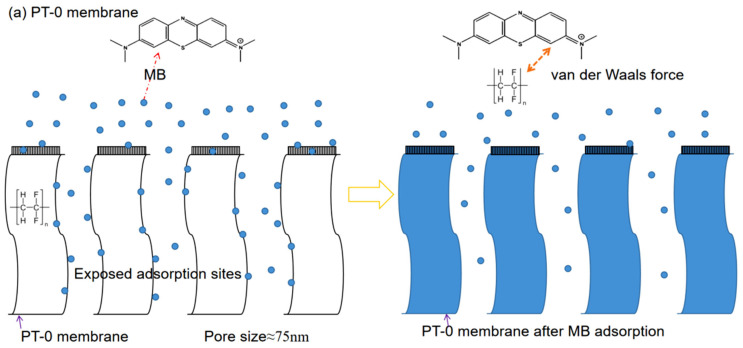

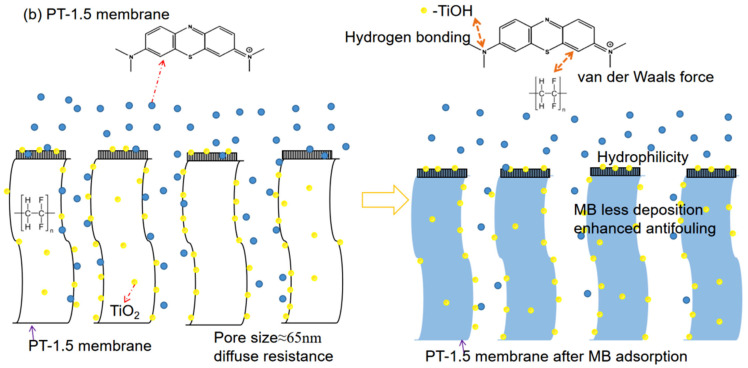
The adsorption mechanism of MB on the PT-0 membrane (**a**) and the PT-1.5 membrane (**b**).

Based on the aforementioned results, the adsorption mechanism of MB on the PVDF substrate membrane is illustrated in Figure 6. For the neat PVDF membrane (PT-0), the hydrophobic aromatic rings of MB interact with the PVDF through van der Waals forces. The high porosity of the membrane (58.8 ± 0.6%) provides abundant adsorption sites, enabling monolayer adsorption of MB within the pores. For the PVDF/TiO_2_ membrane (PT-1.5), TiO_2_ interacts with functional groups of PVDF, occupying some adsorption sites. Additionally, the presence of TiO_2_ reduces the membrane pore size and increases the diffusion resistance of MB, leading to decreased adsorption capacity. However, the addition of TiO_2_ enhances the preferential adsorption of the membrane, reducing MB deposition on its surface and the behavior of the dye molecular through the membrane pores. Anti-fouling performance tests (Figure S7, Supplementary File S11) demonstrate that the irreversible fouling ratio of the PT-1.5 membrane (27.8%) is lower than that of the PT-0 membrane (35.3%), while the flux recovery rate of PT-1.5 (72.2%) exceeds that of PT-0 (64.7%). These results confirm that the incorporation of TiO_2_ effectively mitigates the membrane fouling caused by adsorption.

## 4. Conclusions

A low temperature, high initial MB concentration, and neutral pH (pH = 7) favor the adsorption of MB by PVDF-based membranes. The equilibrium adsorption capacity of the neat PVDF membrane (1.518 ± 0.025 mg/g) is significantly higher than that of the PVDF/TiO_2_ membrane (0.189 ± 0.008 mg/g).

The adsorption of MB on both membranes conforms to the pseudo-second-order kinetic model and Langmuir isotherm model. TiO_2_ reduces the adsorption capacity of the hybrid membrane by changing the PVDF hybrid membranes properties and decreasing the membrane pore size.

Under cross-flow filtration conditions (0.1 MPa), enhanced mass transfer increases the adsorption capacities of both membranes by (15–20)% compared to batch experiments. The neat PVDF membrane exhibits a higher mass transfer zone height (2.3 ± 0.1 cm) and adsorption capacity (2.1 ± 0.09 mg/g), while the PVDF/TiO_2_ membrane demonstrates superior antifouling properties (irreversible fouling rate: 18 ± 1.5%).

PVDF-based membranes can treat dye wastewater through a combined process of “adsorptive enrichment of MB followed by subsequent oxidative degradation”. The neat PVDF membrane is suitable for the rapid adsorption of wastewater with high MB concentrations, whereas the PVDF/TiO_2_ membrane is ideal for scenarios requiring long-term operation and high antifouling performance. Future research should focus on MB desorption and membrane regeneration technologies to enable membrane recycling.

## Figures and Tables

**Table 1:** Kinetic parameters of PT membranes obtained from MB adsorption experiments.

Kinetic Model	Parameters	Membrane Samples	
		PT-0	PT-1.5
PFO	*q*_e(exp)_ (mg·g^−1^)	1.518	0.189
	*k*_1_ (min^−1^)	0.954 ± 0.130	0.248 ± 0.011
	*q*_e(cal)_ (mg·g^−1^)	1.459 ± 0.025	0.185 ± 0.002
	Adj. *R*^2^	0.9779	0.9957
PSO	*k*_2_ (g/(mg·min))	0.943 ± 0.061	2.024 ± 0.199
	*q*_e(cal)_ (mg·g^−1^)	1.516 ± 0.009	0.197 ± 0.003
	Adj. *R*^2^	0.9979	0.9931

**Table 2:** Parameters of isothermal adsorption according to Langmuir and Freundlich equations.

Samples	Temperature (K)	Langmuir Parameters			Freundlich Parameters		
		*Q*_0_ (mg·g^−1^)	*K*_L_ (L·mg^−1^)	*R* ^2^	*K*_f_ (mg·g^−1^) (L·mg^−1^)^1/n^	*n*	*R* ^2^
PT-0	298	8.997 ± 0.284	0.1575 ± 0.013	0.9960	1.660 ± 0.125	2.071 ± 0.125	0.9875
	288	14.481 ± 3.994	0.0113 ± 0.0018	0.9949	0.254 ± 0.095	1.313 ± 0.166	0.9486
PT-1.5	298	2.265 ± 0.118	0.2257 ± 0.0324	0.9805	0.563 ± 0.032	2.480 ± 0.138	0.9871
	283	6.726 ± 2.335	0.0111 ± 0.0060	0.9463	0.116 ± 0.058	1.316 ± 0.217	0.9225

**Table 3:** Kinetic parameters obtained from Dubinin–Rasdushrevich model analysis.

Parameters	Membrane Samples	
	PT-0	PT-1.5
*X*_m_ (g·g^−1^)	0.01585	0.00371
*K*	0.0007	0.0006
*R* ^2^	0.995	0.988
*E* (kJ·mol^−1^)	26.73	28.87

**Table 4:** Thermodynamic parameters of PT-0 membrane and PT-1.5 membrane.

Samples	∆*G*^⊖^ (kJ·mol^−1^)				∆*H*^⊖^ (kJ·mol^−1^)	∆*S*^⊖^ (J·mol^−1^·K^−1^)
	283 K	288 K	293 K	298 K		
PT-0	−16.00	−19.60	−23.21	−26.81	188	720.84
PT-1.5	−19.24	−22.07	−24.89	−27.72	140.8	565.51

## Data Availability

All data generated or analyzed during this study are included in this published article.

## Supplementary Materials

The following supporting information can be downloaded at https://www.mdpi.com/article/10.3390/polym18020233/s1, Figure S1: Schematic diagram of UF cross flow filtration experimental setup; Figure S2: Pictures of PT-0 and PT-1.5 after adsorption of MB; Figure S3: Structure of PVDF, TiO2 and TiO2 /PVDF composite; Figure S4: Isothermal adsorption of MB on PT-0 membrane and PT-1.5 hybrid membrane at different temperatures and modelled by of Langmuir isotherm (A) and Freundlich isotherm(B); Figure S5: Normolized FTIR spectra of membranes before and after adsorption of MB; Figure S6: Zeta potential of PT-0 and PT-1.5 hybrid membrane at different pH; Figure S7: Different filtration resistance of PT-0 and PT-1.5 membranes for BSA solution; Table S1: The parameters about neat PVDF (PT-0) and PVDF/TiO2 (PT-1.5) membranes; Table S2: System energy for PVDF, TiO2 and TiO2/PVDF composite models, van der Waals and electrostatic and interaction energies obtained after geometry optimization; Table S3: The adsorption capacity of dyes including MB in the present works.

## Abbreviations and Nomenclatures

The following abbreviations and nomenclatures are used in this manuscript:

Abbreviation	
PVDF	polyvinylidene fluoride
MB	methylene blue
UV	ultraviolet
TIPS	thermally induced phase separation
DMP	dimethyl phthalate
MO	methyl orange
UV	ultraviolet
PFO	pseudo-first-order
PSO	pseudo-second-order
PT-0	neat PVDF membrane
PT-1.5	PVDF/TiO_2_ (28.5/1.5) membrane
PF	permeate flow
Nomenclatures	
*b*	Langmuir isotherm constant, L/mg
*C*	the effluent MB concentration, mg/L (g/L for DR equation)
*C*_0_	the initial MB concentration or the influent MB concentration (column), mg/L
*C*_B_	the effluent MB concentration at the breakthrough point, mg/L
*C*_E_	the effluent MB concentration at the exhaustion point, mg/L
*C*_e_	the MB concentration at equilibrium or the effluent MB concentration (column), mg/L
*E*	the mean free energy of adsorption, kJ/mol
*f*	a parameter measuring the symmetry of the breakthrough curve
*F*_0_	flow rate of MB solution, mL/min
*H*	the bed depth, cm
*h*_Z_	the height of the mass transfer zone, cm
*K*	the constant of the DR equation related to the adsorption energy
*k*_F_, *n*	Freundlich isotherm constant
*K*_R_, *a*_R_, *β*	Redlich–Peterson isotherm constant
*k*_T_	the rate constant of the Thomas model, L·mg^–1^·h^–1^
*k*_1_	the adsorption rate constant of first–order adsorption, L/min
*k*_2_	the rate constant of pseudo-second-order chemisorption, g/(mg·min).
*m*	the adsorbent dosage or the mass of the sorbent, g
*M*w	molecular weight
*q*_B_	the capacity at the breakthrough point, mg/g
*q*_E_	the capacity at the exhaustion point, mg/g
*q*_e_	the amounts of MB adsorbed at equilibrium, mg/g
*q*_t_	the amounts of MB adsorbed at time *t* (min), mg/g
*q*_T_	the total sorption capacity, mg/g
*Q*	the MB adsorbed, mg/g
*Q*_0_	Langmuir constant related to the capacity and energy of adsorption, mg/g
*R*	the gas constant, kJ/(K·mol)
*T*	the temperature, K
*V*	the solution volume or the effluent volume (column), L
*V*_B_	the volume of solution passed up to the breakthrough point, L
*V*_E_	the volume of solution passed up to the exhaustion point, L
*X*	the amount of MB adsorbed per unit weight of adsorbent, g/g
*X*_m_	the adsorption capacity per unit weight of adsorbent, g/g
*θ*	the flow rate, L/h
*ε*	Polanyi potential = *RT*ln(1 + (1/*C*))
