# Supplementary material for: Adsorption of Methylene Blue on PVDF Membrane and PVDF/TiO2 Hybrid Membrane: Batch and Cross-Flow Filtration Studies
Source: Polymers (Basel). 2026 Jan 16;18(2):233. doi: 10.3390/polym18020233 (PMC12846324; doi:10.3390/polym18020233)
Supplement: Supplementary file 1 [file polymers-18-00233-s001.zip › polymers-4045974-supplementary.pdf]

Adsorption of Methylene Blue on PVDF Membrane and PVDF/TiO_2 Hybrid Membrane: Batch and Cross-Flow Filtration Studies

Fengmei Shi [1], Boming Fan [2], Shuqi Ma [2], Hao Lv [2], Chao Lin [2], Jin Ma [3], Wei Jiang [3] and Yuxin Ma [2,3,*]

[1] Heilongjiang Academy of Black Soil Conservation and Utilization, Heilongjiang Academy of Agricultural Sciences, Harbin 150086, China; ocean-water@126.com
[2] College of Civil Engineering, Heilongjiang University, Harbin 150080, China; 2252470@s.hlju.edu.cn (B.F.); 20236137@s.hlju.edu.cn (S.M.); 2242862@s.hlju.edu.cn (H.L.); 18645188510@163.com (C.L.)
[3] Research and Development Centre, Shandong Aisen Water Industry Co., Ltd., Taian 271021, China; majin_as@126.com (J.M.); jiangwei_as@126.com (W.J.)
* Correspondence: oucmyx@126.com; 2010055@hlju.edu.cn

Supplementary file 1

Table S1 The parameters about neat PVDF (PT-0) and PVDF/TiO_2 (PT-1.5) membranes [1]

Membrane		PT–0	PT-1.5
SEM	Cross section	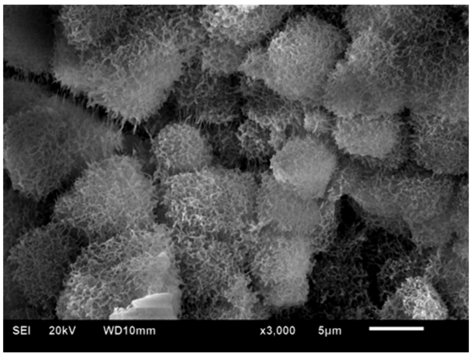	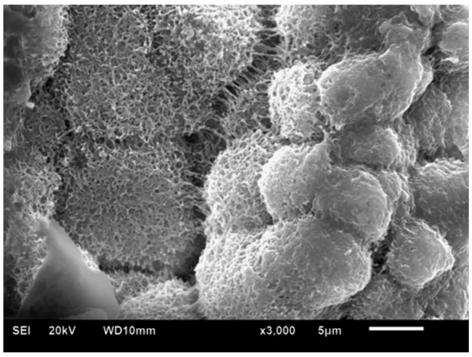
	Upper surface	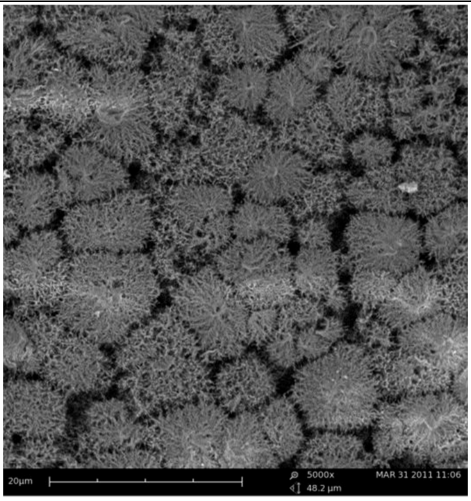	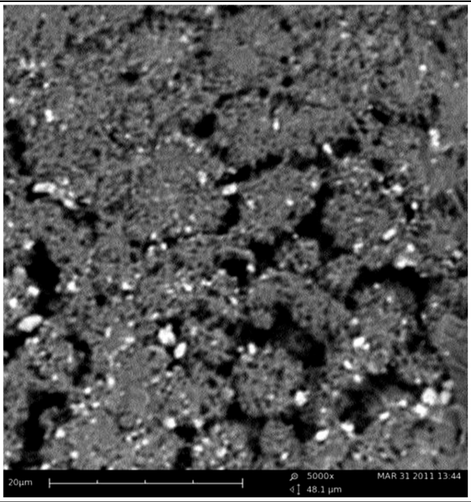
Porosity (%)		58.8±0.6	52.9±0.5
Pure water flux ($L\cdot m^{-2}\cdot h^{-1}$)		63.4±1.3	86.3±3.2
Contact angle (°)		108±2.4	119.5±2.4
Pore size distribution		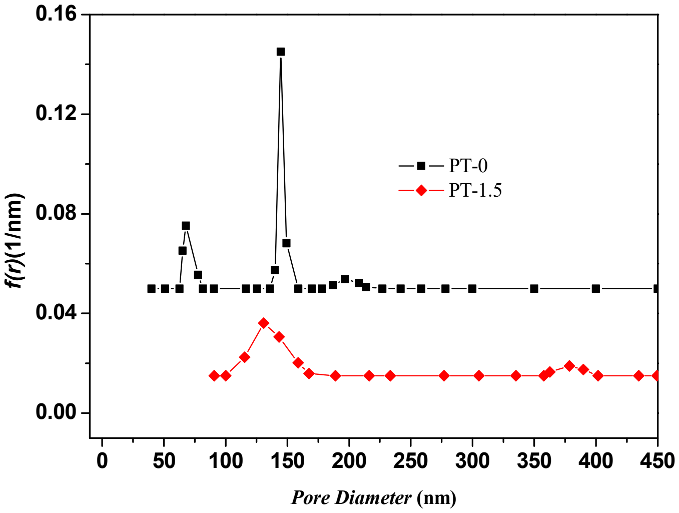	

Supplementary file 2

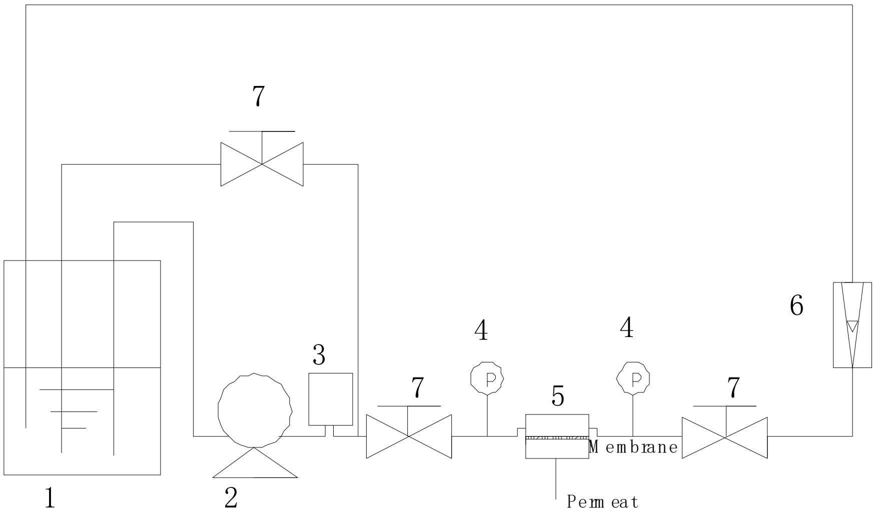

1. Feed tank 2. Booster pump 3. Buffering tank 4. Pressure gauge 5. Flat membrane cell 6. Flowmeter 7. Valve

Figure S1. Schematic diagram of UF cross flow filtration experimental setup [2]

Supplementary file 3

PT-0 and PT-1.5 membranes were immersed in MB solutions for 30min, then washed with distilled water three times. The membranes photographed after 30 min of MB adsorption (C_0=3.68 mg/L, pH=7, T=25°C) using a digital camera (Canon EOS 80D, resolution 6000×4000 pixels). The pictures of PT-0 and PT-1.5 after the membrane treatment were shown in Figure S2.

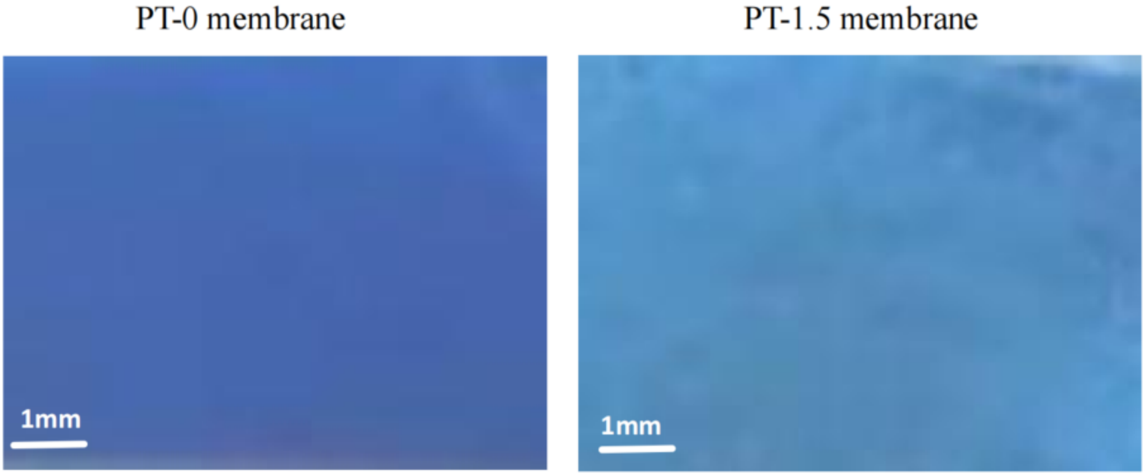

Figure S2. Pictures of PT-0 and PT-1.5 after adsorption of MB

Supplementary file 4

Assuming that the weight and thickness of the membrane sample are the same, they are denoted as *m* and *L*, respectively. The mean pore size R_2 of PVDF/TiO_2 and R_1 of PVDF is 65 nm and 75 nm, respectively. The water weight in the pores of PVDF and PVDF/TiO_2 is mw_1 and mw_2, respectively. The density of water is ρ. The pore number in PVDF and PVDF/TiO_2 is n_1 and n_2, respectively. The pore surface of PVDF and PVDF/TiO_2 is S_1 and S_2 respectively. The porosity of PVDF and PVDF/TiO_2 is 58.8% and 52.9%, respectively.

PVDF : $mw_1/(m+mw_1)=58.8\%$

PVDF/TiO_2: $mw_2/(m+mw_2)=52.9\%$

Then, $mw_1 = 1.427\ m$ and $mw_2 = 1.123\ m$

The pore volume of PVDF: $V_1=mw_1/\rho=n_1\times3.14\times R_1^2\times L$

The pore volume of PVDF/TiO_2: $V_2=mw_2/\rho=n_2\times3.14\times R_2^2\times L$

$S_1=n_1\times2\times3.14\times R_1\times L$

$S_2=n_2\times2\times3.14\times R_2\times L$

$S_1/S_2=(n_1\times R_1)/(n_2\times R_2)$

$S_1/S_2=(mw_1\times R_2)/(mw_2\times R_1)=(1.417\times m\times R_2)/(1.123\times m\times R_1)$

$=(1.262\times R_2)/R_1=1.262\times65/75=1.09$

$(S_2-S_1)/S_1=-8.26\%$

Supplementary file 5

Layered models containing an upper PVDF chain with 20 repeating units and a lower TiO_2 surface were constructed via the following process [3]. Initially, the surface of TiO_2 was built by cleaving the (1 1 0) plane of the three-dimensional anatase TiO_2 cluster，then a super cell was created by increasing the super cell range of U and V to 3 for the three-dimensional TiO_2 cluster and the vacuum thickness was set to 0.0 (Figure S3a) [4] . PVDF chain with 20 repeating units were geometrically optimized (Figure S3b). Afterwards, PVDF chain was added onto the surface of TiO_2 to construct a TiO_2 /PVDF layered model (Figure S3c) . A vacuum slab with a thickness of 30 Å was added to the above PVDF layer so that the PVDF chain only interact with one side of the TiO_2 layer. As an example, the layered model of the PVDF/TiO_2 composite with an initial lattice parameter of 16.0202Å×16.3287Å×52.4112Å is shown in Figure S3c. In a simulation, geometry optimization with an energy convergence tolerance of 0.001 $kcal \cdot mol^{-1}$ and force convergence tolerance of 0.5 $kcal \cdot mol^{-1} \cdot Å^{-1}$ was first carried out to obtain a minimum in the potential energy surface for each cell using a smart algorithm. The maximum number of iterations was 5,000 steps for optimizing procedure to reach the convergence criteria or repeated. After the complete optimization of the structure models, the molecular dynamics (MD) was performed on each suitable model with minimum energy under the following conditions: The canonical NVE ensemble (constant number of particles, volume and total energy) was used at the temperature of 298 K. The dynamic time step was 1.00 fs, and frames were saved every 500 steps.

The van der Waals interactions were calculated using an atom based summation method with a cut-off distance of 12.5Å and the electrostatic interactions were calculated using the Ewald summation method with an accuracy of 0.001 $kcal \cdot mol^{-1}$. All of the simulations were performed using Materials Studio 2019, a commercial molecular simulation software, with condensed-phase optimized molecular potentials for atomistic simulation studies (COMPASS II) with a force field. The interaction energies from optimized models after MD were determined according to Eq. (S1).

$$E_{interaction} = E_{total} - (E_{PVDF} + E_{TiO2}) \quad (S1)$$

According to obtained values of energies (Table S2), it can be shown that the electrostatic interactions and van der Waals can be suggested the dominant ones.

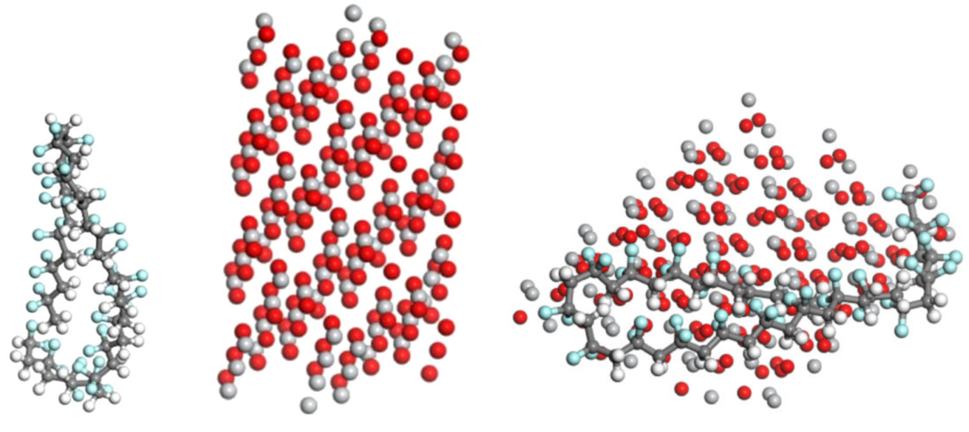

a.PVDF b.TiO_2 c. TiO_2 /PVDF composite

Figure S3. Structure of PVDF, TiO_2 and TiO_2 /PVDF composite (Blue: Fluorine, White: Hydrogen, Black: Carbon,Red: Oxygen, Gray: Titanium).

Table S2. System energy for PVDF, TiO_2 and TiO_2/PVDF composite models, van der Waals and electrostatic and interaction energies obtained after geometry optimization（$kcal{\cdot}mol^{-1}$）.

Energy		E_{total}	E_{PVDF}	E_{TiO2}	$E_{interaction}$
Total energy		-174464.14	-1203.89	-173200.48	-59.77
Valence energy	diag. terms	-311.78	-311.78	0	0.00
	cross terms	-9.98	-9.98	0	0.00
Non-bond energy	Total	-174142.38	-882.12	-173200.48	-59.77
	van der Waals	-136289.69	14.54	-136259.14	-45.09
	Long range correction	-7.15	-0.26	-4.02	-2.87
	Electrostatic	-37845.53	-896.4	-36937.32	-11.81

Supplementary file 6

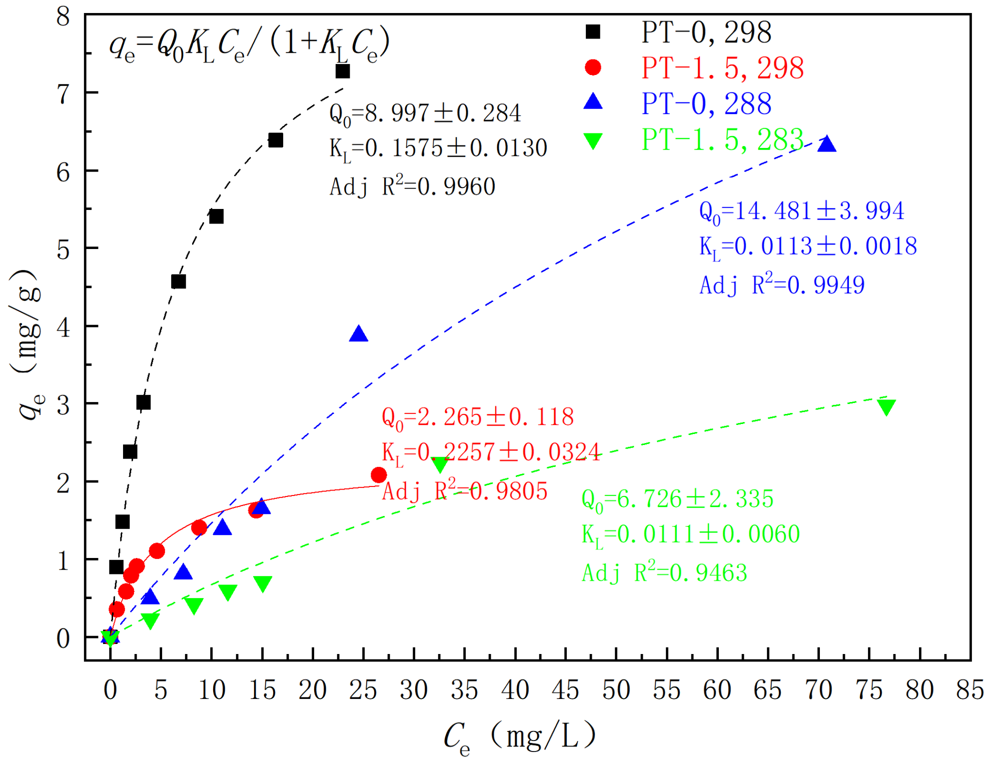

(A) Langmuir isotherm

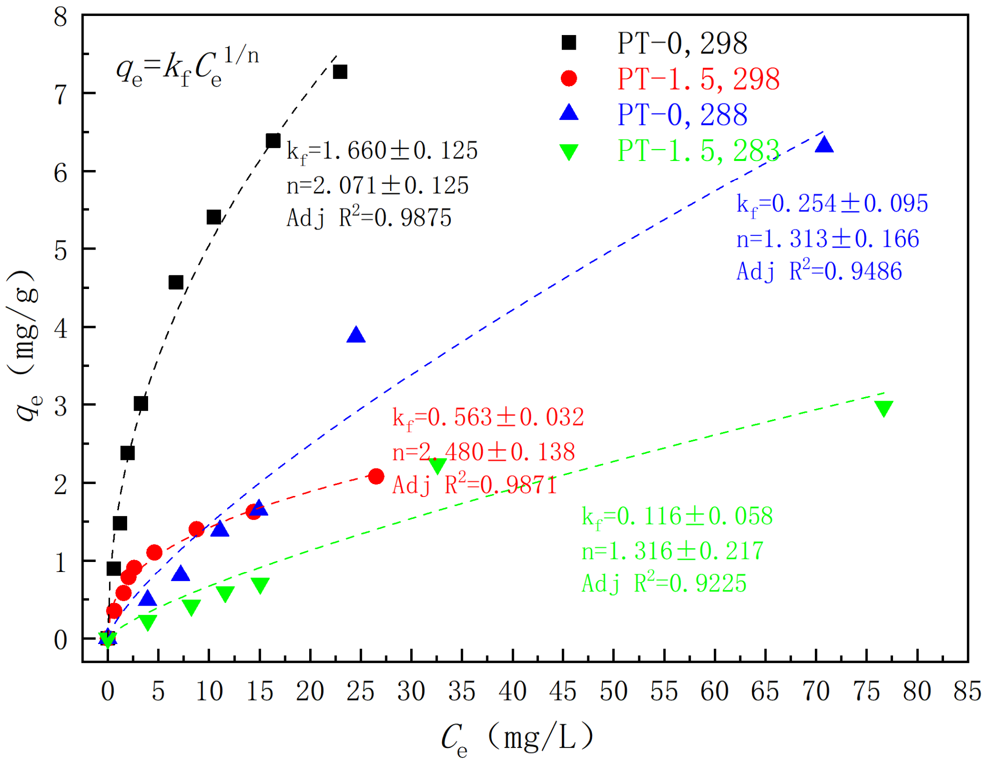

(B) Freundlich isotherm

Figure S4. Isothermal adsorption of MB on PT-0 membrane and PT-1.5 hybrid membrane at different temperatures and modelled by of Langmuir isotherm (A) and Freundlich isotherm(B)

Supplementary file 7

FTIR analysis was performed on a Perkin Elmer Spectrum 2000 spectrometer with a resolution of 1cm^{-1}. Infrared spectra were obtained for membrane samples of PT-0, PT-0MB, PT-1.5 and PT-1.5MB from 4000 to 400 cm^{-1} using attenuated total internal reflection. The part of FTIR from 1800 to 600 cm^{-1} was shown in Figure S5.

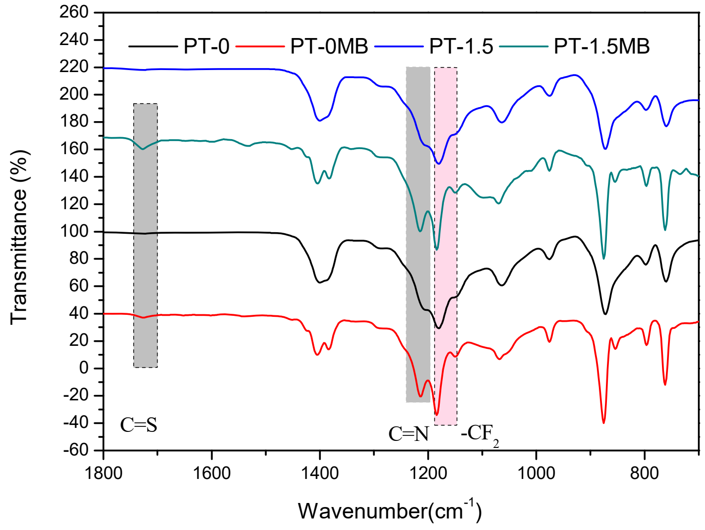

Figure S5. FTIR diagram of PT-0 ,PT-1.5 , PT-0MB and PT-1.5 MB

Supplementary file 8

The Zeta potential with a Zeta potential analyzer (Surpass Anton Paar, Austria). The measurements were conducted in the pH range of approximately 4.0–12. The KCl concentration was 0.001 mol·L^{-1} and the pH was adjusted by adding HCl and KOH solutions. We added the test methods in the supporting materials. The results was shown in Figure S6.

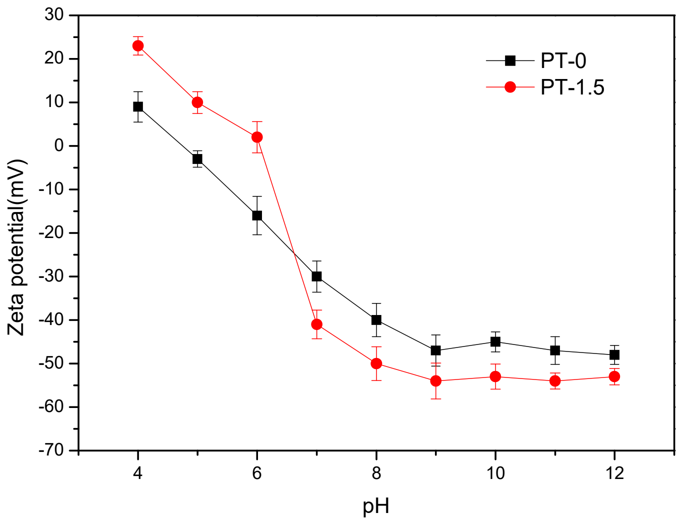

Figure S6. Zeta potential of PT-0 and PT-1.5 hybrid membrane at different pH

Supplementary file 9

For adsorption process, the Gibbs energy can be written by Eq. (S2) and Eq. (S3):

$$\Delta G_{\text{ads}} = \Delta G^{\theta} - RT\ln Q \quad \text{(S2)}$$

$$\Delta G^{\theta} = \Delta H^{\theta} - T\Delta S^{\theta} \quad \text{(S3)}$$

where ΔG_{ads} and $\Delta G^{\ominus}$ are changes in Gibbs free energy ($kJ\cdot mol^{-1}$) of adsorption and at equilibrium, respectively. Q is the reaction quotient at a non-equilibrium system(dimensionless). R is the gas constant ($8.314 J\cdot mol^{-1}\cdot K^{-1}$) and T is the absolute temperature (K). $\Delta H^{\ominus}$ and $\Delta S^{\ominus}$ are changes in enthalpy ($kJ\cdot mol^{-1}$) and entropy ($J\cdot mol^{-1}\cdot K^{-1}$), respectively.

When the adsorption process attains the adsorption equilibrium, $\Delta G_{\text{ads}} = 0$, then:

$$\Delta G^{\theta} = -RT\ln K_{\text{d}} \quad \text{(S4)}$$

where k_{d} is the equilibrium parameter(dimensionless), which can be described as follows [5]:

$$K_{\text{d}} = C^{\ominus} \bullet K_{\text{L}} \bullet 1000 \bullet M_{\text{WMB}} \quad \text{(S5)}$$

where $C^{\ominus} = 1 mol\cdot L^{-1}$, K_{L} is the Langmuir model parameter($L\cdot mg^{-1}$) , 1000， unit conversion($mg\cdot g^{-1}$), M_{WMB} is the molecular weight of MB($g\cdot mol^{-1}$).

Considering Eq. (S4) and Eq. (S5), then:

$$\ln(K_{\text{d}}) = \Delta S^{\theta} / R - \Delta H^{\theta} / RT \quad \text{(S6)}$$

Supplementary file 10

Table S3. The adsorption capacity of dyes including MB in the present works

Polymer	Additive (wt%)	Nanoparticle	Dye	Initial concentration	Adsorption capacity	Reference
PVDF	Dialdehyde cellulose and polyethyleneimine	0.025g	methyl blue	100 mg·L^{-1}	32.1 mg·g^{-1}	6
PVDF	Europium decorated TiO_2	0	methylene orange	3mg·L^{-1}	3.5mg·g^{-1}	7
		0.14%			4.8 mg·g^{-1}	
		13.33%			2.2 mg·g^{-1}	
		26.32%			4.7 mg·g^{-1}	
PVDF	Carboxylated MWCNT	1wt% of casting soultion	methylene blue	30mg·L^{-1}	4.4 mg·g^{-1}	8
PVDF	MIL- 68	0	methylene blue	10mg·L^{-1}	12.66 μg·cm^{-2}	9
		1			21.57 μg·cm^{-2}	
		2			32.7 μg·cm^{-2}	
		4			67.7 μg·cm^{-2}	
		6			74.37 μg·cm^{-2}	
PES	0	0	methylene blue	4.0mg·L^{-1}	3.5mg·g^{-1} at pH =6<br>5.2mg·g^{-1} at pH =9	10
PVDF	Graphitic carbon nitrides	15.75%	rhodamine B	2.0 mg·L^{-1}	30.76 mg·L^{-1}	11
PVDF	Zeolitic imidazolate framework-8	---	methylene blue	20mg·L^{-1}	69.75 mg·g^{-1}	12
PVDF	0	0	methylene blue	3.68 mg·L^{-1}	9.00 mg·g^{-1}	This work
PVDF	TiO_2	1.5 wt%	methylene blue		2.27mg·g^{-1}	This work

Supplementary file 11

Membrane fouling is an inevitable issue encountered during membrane utilization. Bovine Serum Albumin (BSA) is frequently employed as a model protein to systematically investigate the fouling behavior of ultrafiltration/microfiltration (UF/MF) membranes [13]. In this study, BSA was also utilized to assess the impact of TiO_2 introduction on protein fouling of the membrane surface. The filtration experiment was conducted using a protein solution (1000ppm BSA) in a phosphate buffer solution (pH =7.36) to obtain the membrane fouling data.

The fouling experiment procedure was conducted in accordance with the methodology outlined by Kang et al. [14]. Utilizing a cross-flow filtration experimental setup, these experiments were executed under a consistent transmembrane pressure (*TMP*=0.1 MPa). Each membrane underwent compaction using deionized water until a constant permeate flux was achieved, at which point the pure water flux was documented. Subsequently, the reservoir was drained and refilled with the contaminant solution, allowing for the measurement of time-varying permeate flux. Following this, the membrane was cleansed with deionized water to assess flux recovery. The extent of membrane fouling was quantitatively determined employing the resistance in series model, as detailed in the following equation [15,16]:

$$R_t = \frac{TMP}{\mu_{\mathrm{p}} J_{\mathrm{fw}}} = R_{\mathrm{m}} + R_{\mathrm{c}} + R_{\mathrm{ir}} \qquad \text{(S7)}$$

where J_{fw} represents the steady state permeation flux ($m^3 \cdot m^{-2} \cdot s^{-1}$) and *TMP* denotes the transmembrane pressure (Pa), μ_{p} signifies the viscosity of permeate (Pa·s) measured in distilled water after the fouling of the membrane and R_{t} stands for the total filtration resistance (m^{-1}). It is postulated that the total filtration resistance comprises the sum of intrinsic membrane resistance (R_{m}), cake resistance (R_{c}) provided by the loosely bound protein (BSA) layer formed on the membrane surface, irreversible fouling resistance (R_{ir}) contributed by foulant adsorption and strong attachment of foulants such as pore blocking, cake, gel, and biofilm. These are challenging to remove through physical control methods, along with inner pore plugging. Fouling resistance (R_{f}) is defined as the sum of R_{if} and R_{c}. These resistances can be computed from experimental data using the following Eqs. (S8)–(S11):

$$R_{\mathrm{m}} = \frac{TMP}{\mu_{\mathrm{p}} J_{\mathrm{w}}} \qquad \text{(S8)}$$

$$R_{\mathrm{c}} = R_{\mathrm{t}} - \frac{TMP}{\mu_{\mathrm{p}} J_{\mathrm{rw}}} \qquad \text{(S9)}$$

$$R_{\mathrm{ir}} = R_{\mathrm{t}} - R_{\mathrm{m}} - R_{\mathrm{c}} \qquad \text{(S10)}$$

$$R_{\mathrm{f}} = R_{\mathrm{ir}} + R_{\mathrm{c}} \qquad \text{(S11)}$$

where J_{w} represents the clean membrane water flux at a steady state, while J_{rw} denotes the water flux of the fouled membrane, measured after the removal of loosely bound protein by rinsing the membrane in water.

The membranes contaminated with BSA solution were cleansed using a 0.3 wt% NaOH

aqueous solution at a pressure of 0.1MPa for a duration of 10 minutes. Subsequently, they were rinsed with distilled water at the same pressure, and the fluxes (J_{nw}) were measured. The ratio (J_{nw}/J_w) was then calculated.

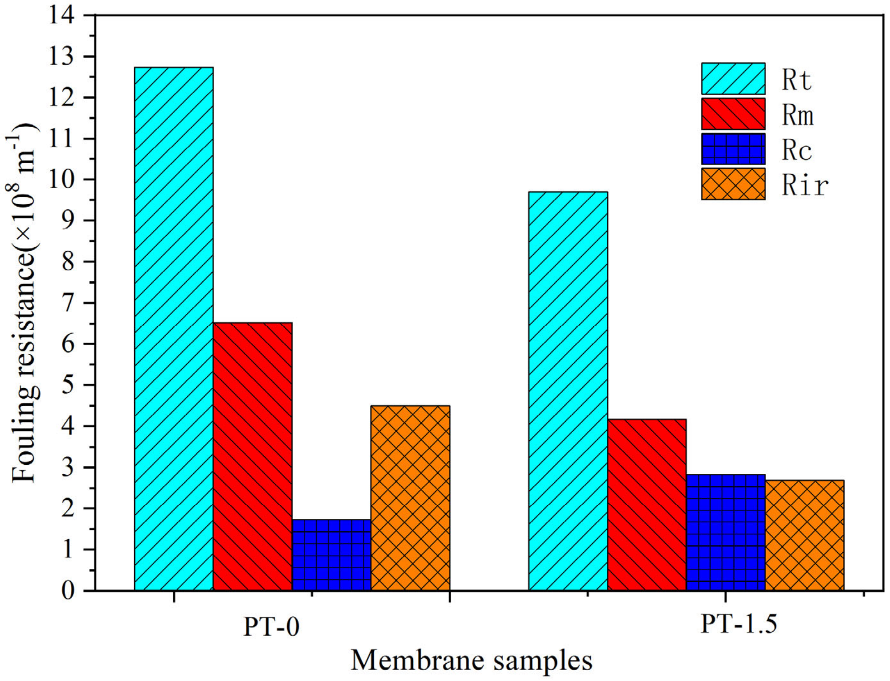

Figure S7. Different filtration resistance of PT-0 and PT-1.5 membranes for BSA solution

References

1. Shi, F., Ma, Y., Ma, J., Wang, P., Sun, W., 2012. Preparation and characterization of PVDF/TiO_2 hybrid membranes with different content of nano-TiO_2. Journal of Membrane Science 389, 522–531.
2. Ma, Y.; Shi, F.; Ma, J.; Wu, M.; Zhang, J.; Gao, C. Effect of PEG Additive on the Morphology and Performance of Polysulfone Ultrafiltration Membranes. Desalination 2011, 272, 51–58.
3. Lazaratou, C.V., Papoulis, D., Vayenas, D.V., Pospísil, M., 2024. Molecular simulation approach for NO_3^--N and NH_4^+-N sorption and desorption in the pores of palygorskite and sepiolite clay minerals. Applied Clay Science 254, Article 107371.
4. Diebold, U., 2003. The surface science of titanium dioxide. Surface Science Reports 48, 53–229.
5. Chen, T., Ma, Y., 2021. Reasonable calculation of the thermodynamic parameters from adsorption equilibrium constant. Journal of Molecular Liquids 322, Article 114980.
6. Liu, J.L., Huang, Y.X., Zhang, G.W., Wang, Q.H., Shen, S.S., Liu, D.P., Hong, Y.L., Wyman, I., 2024. Dialdehyde cellulose (DAC) and polyethyleneimine (PEI) coated polyvinylidene fluoride (PVDF) membrane for simultaneously removing emulsified oils and anionic dyes. Journal of Hazardous Materials 471, Article 134341.
7. Wang, J., Pi, H.M., Zhao, P.C., Zhou, N., 2024. Efficient removal of methyl orange and ciprofloxacin by reusable Eu-TiO_2/PVDF membranes with adsorption and photodegradation methods. RSC Advances 14, Article 18432.
8. Huan, Y.Y., Li, Z.P., Li, C.Q., Li, G.F., 2019. Adsorption performances of methylene blue by poly(vinylidene fluoride)/MWCNT membranes via ultrasound-assisted phase inversion method. Desalination and Water Treatment 163, 83–95.
9. Tan, Y.M., Sun, Z.Q., Meng, H., Han, Y.D., Wu, J.B., Xu, J.L., Xu, Y., Zhang, X., 2019. A new MOFs/polymer hybrid membrane: MIL-68(Al)/PVDF, fabrication and application in highly efficient removal of p-nitrophenol and methylene blue. Separation and Purification Technology 215, 217–226.
10. Zheng, L.L., Su, Y.L., Wang, L.J., Jiang, Z.Y., 2009. Adsorption and recovery of methylene blue from aqueous solution through ultrafiltration technique. Separation and Purification Technology 68, 244–249.
11. Gharbani, P., Mehrizad, A., 2022. Preparation and characterization of graphitic carbon nitrides/polyvinylidene fluoride adsorptive membrane modified with chitosan for Rhodamine B dye removal from water: Adsorption isotherms, kinetics and thermodynamics. Carbohydrate Polymers 277, Article 118860.
12. Liu, Y.F., Zhao, S.Y., Xu, C.G., Huang, J.X., Guo, Z.G., 2024. Construction of ZIF-8 composited hydrogel coated PVDF membrane with durable superhydrophilicity and dyes adsorption properties for efficient separation of emulsified and dyed wastewater. Separation and Purification Technology 353, Article 128299.
13. Damodar, R. A.,You, S.-J., Chou, H.-H., 2009. Study the self cleaning, antibacterial and photocatalytic properties of TiO_2 entrapped PVDF membranes. Journal of Hazardous Materials 172, 1321–1328.
14. Kang, G., Yu, H., Liu, Z., Cao, Y., 2011. Surface modification of a commercial thin film composite polyamide reverse osmosis membrane by carbodiimide-induced grafting with poly(ethylene glycol) derivatives. Desalination 275, 252–259.

15. Bae, T.H., Tak, T.M., 2005. Preparation of TiO_2 self-assembled polymeric nanocomposite membranes and examination of their fouling mitigation effects in a membrane bioreactor system, Journal of Membrane Science 266, 1–5.
16. Choi, H., Zhang, K., Dionysiou, D.D., Oerther, D.B., Sorial, G.A., 2005. Effect of permeate flux and tangential flow on membrane fouling for wastewater treatment. Separation and Purification Technology. 45, 68–78.
